# Cross-modality transfer learning with knowledge infusion for diabetic retinopathy grading

**DOI:** 10.3389/fmed.2024.1400137

**Published:** 2024-05-14

**Authors:** Tao Chen, Yanmiao Bai, Haiting Mao, Shouyue Liu, Keyi Xu, Zhouwei Xiong, Shaodong Ma, Fang Yang, Yitian Zhao

**Affiliations:** ^1^Cixi Biomedical Research Institute, Wenzhou Medical University, Ningbo, China; ^2^Institute of Biomedical Engineering, Ningbo Institute of Materials Technology and Engineering, Chinese Academy of Sciences Ningbo, China

**Keywords:** ultra-wide-field image, domain adaptation, diabetic retinopathy, lesion segmentation, disease diagnosis

## Abstract

**Background:**

Ultra-wide-field (UWF) fundus photography represents an emerging retinal imaging technique offering a broader field of view, thus enhancing its utility in screening and diagnosing various eye diseases, notably diabetic retinopathy (DR). However, the application of computer-aided diagnosis for DR using UWF images confronts two major challenges. The first challenge arises from the limited availability of labeled UWF data, making it daunting to train diagnostic models due to the high cost associated with manual annotation of medical images. Secondly, existing models' performance requires enhancement due to the absence of prior knowledge to guide the learning process.

**Purpose:**

By leveraging extensively annotated datasets within the field, which encompass large-scale, high-quality color fundus image datasets annotated at either image-level or pixel-level, our objective is to transfer knowledge from these datasets to our target domain through unsupervised domain adaptation.

**Methods:**

Our approach presents a robust model for assessing the severity of diabetic retinopathy (DR) by leveraging unsupervised lesion-aware domain adaptation in ultra-wide-field (UWF) images. Furthermore, to harness the wealth of detailed annotations in publicly available color fundus image datasets, we integrate an adversarial lesion map generator. This generator supplements the grading model by incorporating auxiliary lesion information, drawing inspiration from the clinical methodology of evaluating DR severity by identifying and quantifying associated lesions.

**Results:**

We conducted both quantitative and qualitative evaluations of our proposed method. In particular, among the six representative DR grading methods, our approach achieved an accuracy (ACC) of 68.18% and a precision (pre) of 67.43%. Additionally, we conducted extensive experiments in ablation studies to validate the effectiveness of each component of our proposed method.

**Conclusion:**

In conclusion, our method not only improves the accuracy of DR grading, but also enhances the interpretability of the results, providing clinicians with a reliable DR grading scheme.

## 1 Introduction

Diabetic Retinopathy (DR), a typical fundus disease caused by the high level of blood glucose and high blood pressure, is one of the leading causes of visual impairment and blindness (1). The severity of DR can be classified into five stages based on the presence and quantity of retinal lesions, including microaneurysms (MAs), hemorrhages (HEs), soft exudates (SEs), and hard exudates (EXs). These stages encompass normal, mild non-proliferative DR (NPDRI), moderate non-proliferative DR (NPDRII), severe non-proliferative DR (NPDRIII), and proliferative DR (PDR). Accurate grading of DR severity assumes pivotal importance as it guides clinicians in devising personalized treatment strategies. However, the precise determination of DR severity levels can be a time-consuming task for ophthalmologists and presents a formidable challenge for novice ophthalmology residents. Therefore, the development of an automated system for early detection and severity grading of DR holds immense potential, offering substantial benefits to both patients and ophthalmologists alike.

**Figure 1:**
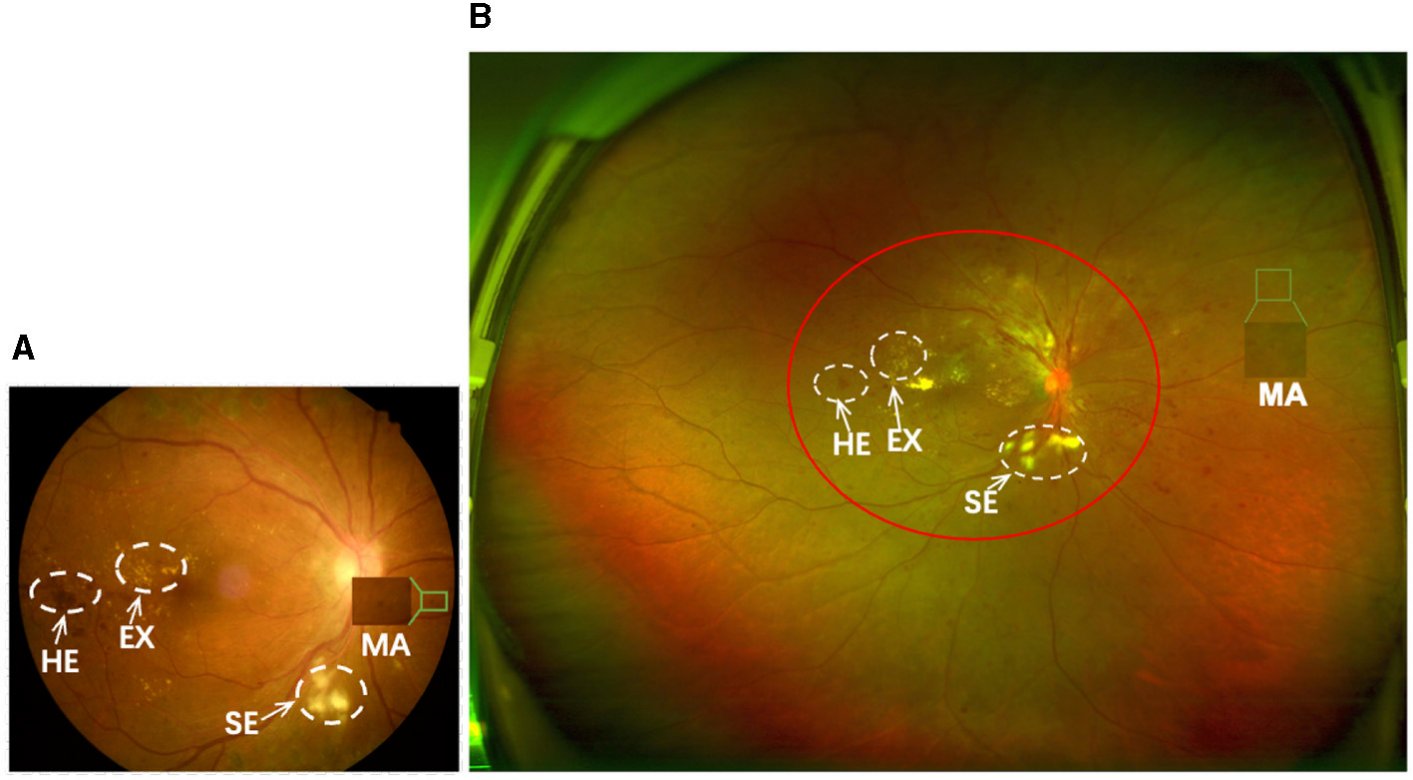
**(A, B)** are samples of CFP and UWF with DR, respectively. The imaging area of **(A)** is approximately that of the red circle in **(B)**. Both can show important lesions associated with DR, but **(B)** gives a more complete picture of the retinopathy.

Over the past half-century, the diagnosis of DR has predominantly relied on the utilization of Color Fundus Photography (CFP), as illustrated in Figure 1A, wherein critical retinal lesion anomalies are depicted. CFP serves as a reasonably effective screening tool for early-stage DR. Nevertheless, CFP exhibits a limited imaging range, typically spanning from 30° to 60°, thereby posing challenges in the identification of anomalies beyond this range. This limitation results in less ideal automated DR grading results.

Optos Ultra-Wide-Field (UWF) imaging technology is a novel non-invasive imaging method with a high resolution and short acquisition durations of 0.25 s. Compared to CFP images, UWF images exhibit a wide imaging range of up to 180°−200°, covering approximately 80.0% of the retina in a single frame (2, 3). This enables UWF images to more effectively detect peripheral retinal lesions (4, 5), as shown in Figure 1B. This enables UWF imagesto hold more advantage in diagnosing DR in comparison to CFP images (6–10). Thus, developing an automated DR grading algorithm based on UWF images is more meaningful.

Over the last decade, methods for automatic screening or grading of DR severity using CFP images have been rapidly developed with remarkable accuracy of ≥90.0% (11–17). This is largely due to the large scale, high quality CFP dataset that is publicly available, which provide pixel-level annotations and image-level annotations, such as EyePACS (18), DDR (18), IDRiD (19) etc. Despite several studies (20, 21) have conducted DR grading using UWF images, the performance of these methods has been found to be less satisfactory compared to those using the CFP iamges. The reasons may be attributed to the following factors: (1) The scarcity of large-scale annotated data for deep learning training in UWF imaging poses a significant challenge in training high-performing grading models using fully supervised methods. The only public available dataset of UWF contains 256 UWF images with DR (22). (2) The lesion information is crucial for enhancing the precision of DR grading. However, the contrast divergence between lesions and ordinary tissue in UWF images is slight, which hampers precise grading of DR.

To address these challenges, we aim to utilize a substantial dataset of well-annotated CFP images along with knowledge infusion to enhance the performance of DR grading. Recent studies have explored unsupervised domain adaptation learning methods to mitigate the domain-shift issue between the source and target domains (23–25). These methods leverage external labeled datasets to acquire general knowledge of diseases and transfer this knowledge to object categories without labels. In this study, we design a transfer learning model utilizing the rich pixel-level and image-level annotations available in CFP images to facilitate the DR grading in UWF images. A preliminary version of this work has been previously published in conference proceedings (26). In this paper, we present the following extensions:

1) To enhance the recognition of complex lesions for the lesion segmentation task, we introduce a novel roll-machine modulated feature fusion block. To enable comprehensive evaluation, we construct a new dataset called UWF-seg, which includes 27 images with annotations of different lesions. We provide evaluations on UWF-seg and additional result analyses to further validate the effectiveness of our proposed method.

2) To gain deeper insights into proposed method, we conduct extensive additional experiments, including evaluations with a larger set of unlabeled images, exploration of different loss weights, and analysis of different exemplar images. Moreover, we carefully examine failure cases to identify potential limitations for improvement.

3) We enrich the discussion in this study by providing a more comprehensive analysis of the relationship and comparison between our work and related studies. Additionally, we offer a detailed technical description of our proposed method and engage in an in-depth discussion of its limitations. Finally, we outline future research directions to address these limitations and extend the scope of our work.

## 2 Related works

### 2.1 Computer-aided diagnosis in UWF

In this section, we survey the current studies that utilizes UWF imaging to identify a range of retinal diseases, with a particular emphasis on the computer-aided diagnosis of diabetic retinopathy. Recently, deep learning models have been applied to UWF images with the goal of detecting various retinal diseases. For instance, central retinal vein occlusion (27, 28), Sickle cell retinopathy (29, 30) and retinal detachments (31, 32), respectively. These studies have underscored the clinical advantages of employing UWF imaging in diagnosing various peripheral retinal pathologies. Nagasawa et al. (33) conducted a study to assess the accuracy of utilizing UWF fundus images alongside the VGG16 model for detecting PDR. In a subsequent investigation (34), they extended their research by comparing the accuracy of VGG16 using two distinct types of retinal images for DR grading. These methodologies primarily concentrate on the binary classification of DR, placing a premium on practical clinical relevance over architectural enhancements in network design. In efforts to refine the precision of DR grading, Liu et al. (35) curated a proprietary UWF dataset comprising 101 DR fundus images. They devised a deep learning-based automatic classification model integrating a novel preprocessing technique, achieving an average accuracy of 0.72. However, the utilization of UWF imaging in detecting DR-related lesions remains relatively underexplored, with only a few researchers delving into this domain. For example, Levenkova et al. (36) utilized support vector machine (SVM) algorithms to identify features of DR lesions, categorizing them into bright lesions (such as cotton wool spots and exudates) and dark lesions (including microaneurysms, spots, and flame-shaped hemorrhages). However, their study exclusively focused on segmenting bright and dark signs, neglecting the comprehensive diagnosis of DR grade. The efficacy of these methodologies in addressing DR challenges largely hinges on the availability of meticulously annotated data. Nevertheless, the scarcity of UWF data and the prohibitive costs associated with labeling pose significant barriers, thus constraining access to this valuable resource and hindering the broader implementation of deep learning techniques in this domain.

Furthermore, many current learning-based methods for grading DR lack interpretability and fail to integrate prior knowledge to inform the classification process. Thus, there is a critical need to develop an interpretable approach for DR grading using UWF images in an unsupervised manner, capitalizing on inherent lesion features. In particular, Ju et al. (7) introduced a methodology that incorporates CFP images to aid in training diagnostic models based on UWF images. They utilized an enhanced CycleGAN framework to bridge the domain disparity between CFP and UWF images, thereby generating new data with UWF image characteristics. Subsequently, these generated images underwent labeling via pseudo-labeling techniques. While the model exhibited promising performance across various retinal disease diagnosis tasks, including DR grading, its reliance primarily on a GAN-based model for transforming CFP images into UWF fundus images is notable. This strategy aimed to augment the limited UWF imaging dataset with additional data. However, the approach encountered challenges in effectively transferring knowledge from CFP images to UWF images. Consequently, the model's performance remains susceptible to the potential impact of synthesized UWF images.

### 2.2 Domain adaptation

Domain adaptation (DA) serves as a crucial paradigm within the realm of transfer learning in machine learning, aimed at mitigating the distribution disparity between domains. Fundamentally, it involves identifying similarities between different data distributions in related tasks and harnessing these similarities to facilitate cross-domain recognition problems (37–39). Several systematic reviews (40–42) offer comprehensive insights into this method from various perspectives. For instance, domain adaptation from general to complex situations, including methods based on domain distribution difference (43, 44), adversarial learning (45, 46), reconstruction-based methods (47, 48), and sample generation-based methods (49, 50). Recently, the efficacy of DA leveraging deep architecture has garnered empirical support across numerous vision tasks, including textual emotion (51), object detection (52), and pose estimation (53). Unsupervised domain adaptation (UDA) represents a notable advancement, facilitating the prediction of target domain data without necessitating manual annotation (43). This approach offers a potential and viable avenue for mitigating the challenges associated with limited labeled data.

In the realm of medical image analysis, Unsupervised Domain Adaptation (UDA) stands as a widely explored area aimed at mitigating disparities between cross-domain datasets derived from various imaging equipment types, thereby enhancing image segmentation or classification. Kamnitsas et al. (54) introduced UDA techniques to biomedical imaging, presenting an unsupervised domain-adaptive network tailored for brain lesion segmentation. Furthermore, Chai et al. (55) delved into the potential of reducing disparities between Optical Coherence Tomography (OCT) images captured using Topcon and Nidek devices, with the aim of achieving more effective segmentation of the choroid region. Due to the substantial scarcity of data in certain intricate medical image tasks, there has been widespread interest in employing unsupervised transfer learning to alleviate data constraints, leading to notable advancements as evidenced by works (24, 56, 57). Zhang et al. (58) introduced a cooperative UDA algorithm tailored for microscopy image disease diagnosis, demonstrating that the integration of rich labeled data from relevant domains can effectively enhance learning in cross-domain detection tasks. In the domain of DR grading, the predominant focus has been on the transition between DR lesion detection and grading tasks (59–61). However, these approaches have primarily been developed based on conventional color fundus images. In our prior investigation (26), we explored the application of UDA to train a diagnostic model for UWF images, leveraging the assistance of CFP images. Our experimental findings demonstrated that the proposed method effectively transfers knowledge from CFP images pertaining to DR to UWF images, consequently leading to enhanced performance in DR disease recognition tasks.

## 3 Proposed method

### 3.1 Problem formulation

**Figure 2:**
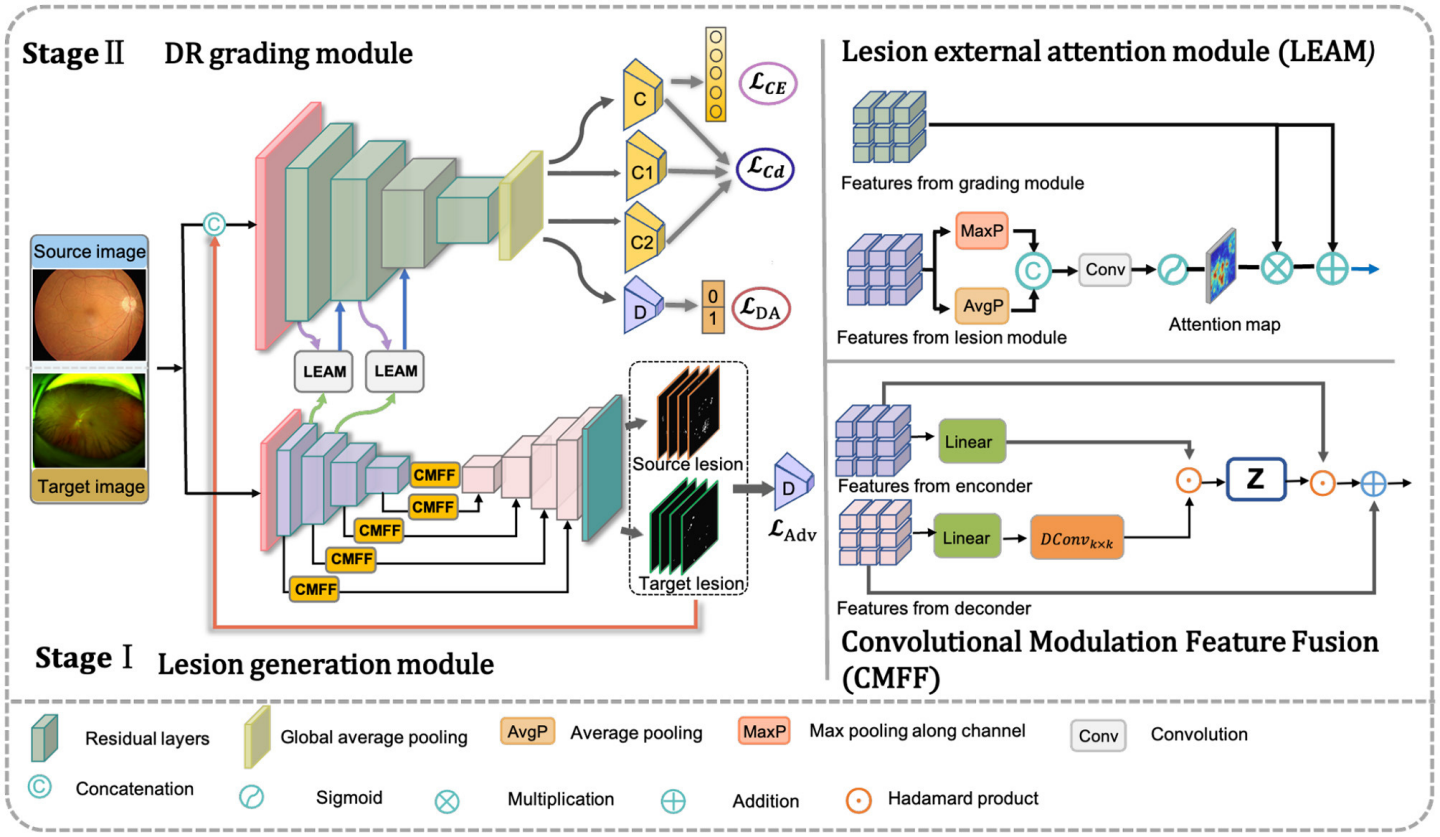
Framework of the proposed method. Our net includes three components: the DR grading module based on transfer learning, adversarial multi-lesion masks generate network, and lesion external attention module. The input data consists of a very small set of unlabeled target images and a large set of annotated source images. The adversarial multi-lesion masks generate network is used to learn multi-lesion masks, where target lesion predicted will with the original images as inputs for training DR grading module based on transfer learning. At the same time, the lesion external attention module aims to force classification network to pay those lesions for improving the final disease grading performance.

Given annotated color fundus photography (CFP) images *X*^*S*^ as the source domain and ultra-widefield (UWF) images without any annotations *X*^*T*^ as the target domain, our objective is to leverage the high-quality annotated CFP images to train a robust diabetic retinopathy (DR) grading model for UWF images in an unsupervised manner. Additionally, we incorporate a lesion segmentation model *G*(·) to augment the grading model *C*(·) with extra knowledge, mirroring the clinical process of assessing DR severity and enhancing grading accuracy. To train the segmentation model, our aim is to minimize the disparity between the predicted lesion maps from UWF images and the ground truth lesion maps from CFP images, as formulated by the following objective function (Equation 1):


(1) $$\begin{array}{l} \underset{G}{\min}\overset{L}{\underset{l=1}{\sum }}L_{Seg}\left(G\left(X^{S}\right),G\left(X^{T}\right),s_{l}^{S},s_{l}^{T}\right) \end{array}$$

where $s_{l}^{S}$ denotes the the CFP lesion maps of pixel-level annotated CFP images and $s_{l}^{T}$ is the UWF predicted lesion maps. L is the total number of lesion varieties related to a particular disease. The optimization function for the disease grading model is defined as Equation 2:


(2) $$\underset{C}{\min}{ℒ}_{Cls}\left(C\left(X^{T}+G(X^{T})\right)\cdot \mathrm{LEAM}\left(G\left(X^{T}\right)\right),y_{c}{\;}^{I}\right)$$

where $y_{c}^{I}$ denotes the disease severity classification prediction for image-level annotated CFP data. Thus, the pivotal aspect in achieving collaborative learning across different modules lies in the design and optimization of *G*(·), *C*(·), and *LEAM*(·). The overall architecture of the proposed framework is illustrated in Figure 2.

### 3.2 Unsupervised DR grading module

The DR grading module comprises a deep feature extractor *F*_*E*_(·), a label predictor *C*, and a domain predictor *D*, facilitating unsupervised domain adaptation for knowledge transfer. Meanwhile, to enhance the extraction of discriminative features tailored for diabetic retinopathy (DR) classification, we employ two classifiers, *C*_1_ and *C*_2_. These classifiers aid the feature extractor in disregarding domain differences. Given the complexity of domain adaptation evaluation, we employ the pretrained ResNet50 encoder (62) in the hierarchical module. Compared to ResNet128 and ResNet32, ResNet50 has moderate depth and parameter count, making it easier to train and fine-tune for feature extraction. Thus, it can extract *n*-dimensional feature vectors, denoted as *f*^*S*^ and *f*^*T*^, corresponding to the source and target domains, respectively.

Subsequently, a class label predictor *C* and a domain predictor *D* follow. The label predictor estimates the probability of DR severity grading, while the domain predictor ensures learned feature invariance across domains. The feature vector *f* is mapped to *d* = 0 (for input from the source domain *S*) or *d* = 1 (for input from the target domain *T*) by the domain predictor, ensuring similar feature distributions across domains. The domain predictor D comprises two fully connected (FC) layers. The first FC layer is accompanied by batch normalization (BN) and a ReLU activation function, while the second layer is followed by BN and a softmax activation function. The feature vector f is transformed by D into either d=0 (when the input is *X*^*S*^ or d=1 (when the input is *X*^*T*^), ensuring that the feature distributions from both domains remain as similar as possible. While the domain predictor effectively achieves domain alignment, it may not guarantee class discriminability. To ensure discriminative feature representations, we maximize the discrepancy between the two classifiers, *C*_1_ and *C*_2_, to obtain highly discriminative features. The details of the loss function are as follows in Equation 3:


(3) $$\begin{array}{l} {ℒ}_{cd}=E_{x_{j}^{t}\sim D_{t}}{\left\|C_{1}\left(G\left({\hat{x}}_{j}^{t}\right)\right)-C_{2}\left(G\left({\hat{x}}_{j}^{t}\right)\right)\right\|}_{1}\\ \;+{\left\|C\left(G\left({\hat{x}}_{j}^{t}\right)\right)-C_{1}\left(G\left({\hat{x}}_{j}^{t}\right)\right)\right\|}_{1}\\ \;+{\left\|C\left(G\left({\hat{x}}_{j}^{t}\right)\right)-C_{2}\left(G\left({\hat{x}}_{j}^{t}\right)\right)\right\|}_{1} \end{array}$$

*C*, *C*_1_, and *C*_2_ denote three pre-trained classifiers trained via supervised learning on the source domain. When *G* and *C* are fixed, maximizing the discrepancy between *C*_1_ and *C*_2_ in the target domain enables them to identify target samples not captured by the support vectors of the source. By training *G* to minimize this discrepancy, while *C*_1_ and *C*_2_ remain fixed, the resulting target features become highly discriminative. The primary classifier *C* defines a decision hyperplane between *C*_1_ and *C*_2_, optimizing the distance between the support vectors and the decision boundary. It's important to note that the class predictor *C* is utilized during both training and testing procedures to obtain grading labels, while the domain predictor *D*, *C*_1_, and *C*_2_ are only employed during training.

### 3.3 Adversarial lesion segmentation module

To mimic the clinical process of assessing DR severity, we introduce an adversarial domain adaptation (DA)-based UWF segmentation model. This model serves as an ancillary tool for UWF lesion segmentation. A schematic diagram of the lesion segmentation subnet is depicted in the orange section of Figure 2. As illustrated, the framework comprises two primary components: the convolutional modulation-based lesion generator *G*(·) and the adversarial domain discriminator *D*(·). We denote pixel-level lesion annotations as *X*^*S*^, and the target domain data without such annotations as *X*^*T*^. Here, *X*^*S*^ and *X*^*T*^ belong to *R*^*C*×*W*×*H*^, where *H*, *W*, and *C* represent the height, width, and number of channels of the input, respectively. Additionally, *M*^*S*^ and *M*^*T*^ represent the lesion prediction results for the source and target domain data, respectively. The proposed UWF lesion segmentation subnet is elaborated as follows.

#### 3.3.1 The convolutional modulation-based lesion generator

Our proposed model is implemented based on a U-shaped structure, also known as a Res-Unet proposed by Xiao et al. (63). We extended the Res-UNet with the deeper multi-scale residual module and modified it to be a lesion generator. Specifically, the encoder and decoder components for the mask generator comprise nine feature mapping tuples. Additionally, two convolutional layers with Sigmoid activation are appended to generate a lesion mask for the input image. This architecture serves as the segmentation backbone network (**Base**) for the lesion segmentation task.

In addition, we introduce a Convolutional Modulation Feature Fusion block (CMFF) to enhance the model's ability to learn complex lesions and achieve accurate segmentation in a larger receptive field of UWF images. The convolutional modulation operation (64) encodes spatial features to simplify self-attention and can better leverage large kernels (≥7 × 7) nested in convolutional layers. Inspired by U-Transformer (65), we employ multiple CMFF blocks instead of traditional skip connections, aiming to fully integrate multi-scale high-level feature maps with relevant encoding features, as illustrated in Figure 2. A second CMFF block is positioned at the end of the encoder to assimilate distant knowledge from the input image and associate each pixel in the high-level semantic features learned by the encoder. This approach enables the model to capture the receptive field of the entire image and achieve accurate lesion segmentation in UWF images, as depicted in Figure 2. Taking the first CMFF block as an example, for the feature maps *X*_*i*_ and $Y_{i}\in R^{C\times W\times H}$ from the encoder and decoder, respectively, the CMFF operation can be expressed as follows in Equations 4–7:


(4) $$\begin{array}{l} Z_{i}=A_{i}⊙V_{i} \end{array}$$

(5) $$\begin{array}{l} A_{i}=DConv_{k\times k}\left(W_{1}Y_{i}\right) \end{array}$$

(6) $$\begin{array}{l} V_{i}=W_{2}X_{i} \end{array}$$

(7) $$\begin{array}{l} F_{i}=Z_{i}⊙X_{i}⊕Y_{i} \end{array}$$

where ⊙ denotes Hadamard product, *W*_1_ and *W*_2_ are weight matrices of two linear layers, *DConv*_*k*×*k*_ denotes denotes a depthwise convolution with kernel size k × k.

#### 3.3.2 Adversarial domain discriminator

The lesion generator *G*(·) is trained on images with pixel-level lesion annotations from the source domain (CFP images) and unlabeled UWF images from the target domain as input, enabling automatic lesion segmentation. With pixel-level annotated lesion masks *Y*^*S*^ of the source domain, a combination of Dice loss ℒ_*Dice*_ and cross-entropy loss ℒ_*CE*_ is employed to minimize the difference between the predicted lesion map *M*^*S*^ and the ground-truths *Y*^*S*^. The trained *G*(·) is capable of outputting a segmentation result, which represents a structured output containing feature similarity between the source and target domains.

To further transfer knowledge from the source domain to the target domain in the output space, an adversarial domain discriminator *D*(·) needs to be introduced. The primary objective of *D*(·) is to ensure that the generated sample closely resembles real data. In our implementation, we consider the source lesion maps *M*^*S*^ predicted by *G*(·) as the real data branch and the target lesion maps *M*^*T*^ predicted from the UWF data as the fake data branch. By using *M*^*S*^ and *M*^*T*^ as inputs for *D*(·), with an adversarial loss, we aim to reduce the domain gap between the source and target domains, thereby enhancing the accuracy of lesion prediction in the target domain images. The total loss for optimizing the lesion segmentation task can be defined as in Equations 8–10:


(8) $$\begin{array}{l} L_{Total}=L_{Adv}+\lambda L_{Seg}. \end{array}$$

(9) $${ℒ}_{Adv}=\underset{G}{\min}\underset{D}{\max}E[log(D(M^{S})]+E[log(1-D(M^{T})].$$

(10) $$\begin{array}{l} {ℒ}_{Seg}={ℒ}_{Dice}\left(M^{s},Y^{s}\right)+{ℒ}_{CE}\left(M^{s},Y^{s}\right)=\\ \;\sigma \frac{2\times \left|M^{s}\cap Y^{s}\right|}{\left(\left|M^{s}\right|+\left|Y^{s}\right|\right)}+\\ \;E\left[-Y^{s}\cdot \log M^{s}-\left(1-Y^{s}\right)\cdot \log\left(1-M^{s}\right)\right]. \end{array}$$

where λ the balance weight of two objective functions, σ the balance weight of Dice loss and cross-entropy loss.

The domain discriminator consists of four convolutional tuple maps, as illustrated in the Figure 2. Each tuple comprises convolutional operations with varying kernel sizes aimed at progressively encoding contextual information to expand the receptive field. Specifically, the first tuple conducts convolutional operations with a kernel size of 7 × 7 and padding of 3. Subsequently, the second and third tuples perform convolutional operations with a kernel size of 5 × 5 and padding of 2. The final convolutional operation employs a kernel size of 3 × 3 and padding of 1. A stride of 2 is applied for each tuple, with linear ReLU activation and batch normalization also incorporated. The output of the last convolutional layer undergoes spatial dimensionality reduction via an adaptive average pooling layer. Subsequently, a binary output is generated through a fully connected layer and Sigmoid activation function, facilitating the distinction of whether the predicted lesion map output originates from the source domain or the target domain.

#### 3.3.3 Lesion external attention module

Despite the integration of the generated lesion maps with the grading module, the independent nature of the lesion generation module and the grading module hinders the effective utilization of lesion information to guide the learning process of the grading module. Furthermore, the disease grading task is confronted with challenges beyond the diverse lesion types of varying clinical significance. The disease grading task also encounters challenges stemming from complex background artifacts (such as eyelash and eyelid interference) and noise present in ultra-widefield (UWF) images, particularly when employing unsupervised approaches. To improve the integration of filtered lesion knowledge into the grading module, we introduce a Lesion External Attention Module (LEAM). Unlike previous self-attention mechanisms (66), we utilize an external module, specifically the lesion generation module, to generate the lesion attention map. This attention map is subsequently used to re-calibrate the features within the grading module. The LEAM acts as a bridge, facilitating the effective utilization of lesion information obtained from the lesion generation module to guide the learning process of the grading module. This mechanism assists the grading module in a human-like manner for classification, automatically extracting task-specific lesion regions while ignoring irrelevant information to enhance grading accuracy.

The details of LEAM are illustrated in Figure 2. We begin by extracting the feature maps $f_{i}^{L}$ from the lesion generation module, where *i* represents the i-th intermediate layer of the generator *G*_*L*_(·). Max pooling and average pooling are performed across channels to obtain two spatial lesion descriptors. Max pooling helps capture locally important features in the image, while average pooling aids in extracting global features and reducing noise. Combining both enhances feature representation, enabling the model to better understand the image. Subsequently, these concatenated descriptors are fed into a convolutional layer followed by a sigmoid activation layer to generate the lesion attention map.

In our approach, the disease grading module and LEAM are intricately integrated. Initially, we utilize *G*_*L*_(·) to extract the lesion feature maps. Once pre-trained, $f_{l=i}^{L}$ (where *i* denotes the i-th different intermediate base layer of the U-shaped network encoder) serves as input to the LEAM. Following maximum pooling, average pooling, and convolution operations, a lesion attention map $m_{l=i}^{L}$ is produced. Subsequently, we multiply the feature maps $f_{i}^{G}$ from the grading module (with *i* denoting the i-th intermediate layer of the grading module) by $m_{i}^{L}$. This is followed by an element-wise summation operation with $f_{i}^{G}$ to derive the new feature maps ${\tilde{f}}_{i}^{G}$. The overall attention process can be summarized as follows in Equation 11:


(11) $$\begin{array}{l} m_{i}^{L}=\sigma \left(Conv\left(AvgPool\left(f_{i}^{L}\right)∥MaxPool\left(f_{i}^{L}\right)\right)\right),\\ {\tilde{f}}_{i}^{G}=(f_{i}^{G}⊗m_{i}^{L})⊕f_{i}^{G}, \end{array}$$

where ∥ denotes the concatenation operation, σ denotes the sigmoid activation function. ⊗ and ⊕ demote the element-wise multiplication and element-wise sum, respectively. This design allows more multi-scale pathological information to be extracted from UWF images, which helps our unsupervised transfer learning framework to be more accurate and robust.

## 4 Experiments

### 4.1 Data description

In our experiment, two types of datasets were involved: source domain and target domain. A summary of used datasets related to this experiment is provided in Table 1.

**Table 1:** The summary and distribution statistics in our project image datasets.

**Dataset**	**Annotation modes**	**Images**	**Nomal**	**NPDRI**	**NPDRII**	**NPDRIII**	**PDR**	**Tasks**
IDRID	Pixel-level	81	-	-	-	-	-	Seg-Source
EYEPACS	Image-level	8,000	1,715	1,715	1,714	1,514	1,342	Grad-Source
DeepDRiD	Image-level	206	60	57	56	23	4	Grad-Target
Local-UWF	Pixel-level	27	0	6	9	7	5	Seg-Target
		Image-level	1212	412	202	193	218	187	Grad-Target

For the source domain data, publicly accessible datasets with annotations, such as IDRID and EYEPACS, are available. However, for the target domain data, there is currently no publicly available dataset with high-quality lesion segmentation labels. Therefore, one of the primary objectives of our benchmark is to introduce a fine-grained lesion annotated dataset to facilitate a more comprehensive evaluation of the proposed lesion segmentation subnetwork and enable a more interpretable diagnosis of DR. Additionally, we assess the grading performance of our DeepMT-DR method on the public UWF dataset, namely DeepDRiD. Detailed information about existing datasets and our proposed dataset is provided below.

#### 4.1.1 IDRID

IDRID is the DR dataset providing pixel-level multi-lesion annotations, is one of the most commonly used public datasets for DR segmentation tasks. It comprises 81 CFP images depicting DR symptoms, with 54 allocated for training and 27 for testing. Medical experts meticulously annotated four types of lesions–MA (80), HE (80), EX (81), and SE (40)–using binary masks. This dataset serves as the source domain data to train the lesion generator.

#### 4.1.2 EyePACS

EyePACS sourced from the DR Challenge - Kaggle Diabetic Retinopathy Detection Competition,^1^ comprises 88,702 CFP images and offers image-level grading annotations across five categories. To maximize the inclusion of diseased samples, we randomly sampled 8,000 images (approximately 1,600 images per category) from EyePACS, creating a new subset to serve as the source domain for training the grading subnetwork.

#### 4.1.3 DeepDRiD

DeepDRiD is the only DR dataset providing multi-grading annotations, to the best of our knowledge. It contains 256 UWF images with symptoms of DR and is into UWF Set-A (77 patients, 154 images) for training, UWF Set-B (25 patients, 50 images) for testing and UWF Set-C (26 patients, 52 images) for validating. We use the UWF Set-C to evaluate the grading performance of our DeepMT-DR method.

#### 4.1.4 Local UWF

We have compiled a finely annotated Diabetic Retinopathy (DR) Ultra-Widefield (UWF) dataset, comprising two distinct subsets. The first subset, named UWF segmentation subset (UWF-Seg), consists of 27 images annotated with pixel-level lesion labels and image-level grading annotations. Lesion annotations encompass Microaneurysms (MA), Hemorrhages (HE), Exudates (EX), and Soft Exudates (SE), making this subset specifically tailored for evaluating segmentation performance. The second subset, named UWF grading subset (UWF-Grad), comprises 877 images annotated with grading labels by three ophthalmologists, ranging from 0 to 4. During the segmentation sub-network training, UWF-Grad served as the target domain, while UWF-Seg was utilized for testing. For training the grading model, the person-UWF dataset was partitioned into 60% for training and 40% for testing. It is noteworthy that our proposed method underwent training without leveraging any labels.

**Dataset construction:** The UWF image data were mainly collected from local partner hospitals. To fully protect patient privacy, data security regulations was strictly adhered in our dataset construction. All the images were captured by Optos Daytona (P200T) UWF canning laser ophthalmoscope with an imaging resolution of 3900 × 3072 pixels. To ensure data quality and task accuracy, three selection principles were adopted: 1. Removal of images with quality issues and non-standard imaging; 2. Deletion of images with severe blurriness; 3. Prioritization of images without laser treatment. For the UWF-seg dataset, images with higher severity of diabetic retinopathy and a greater diversity of lesion types were selected.

**Figure 3:**
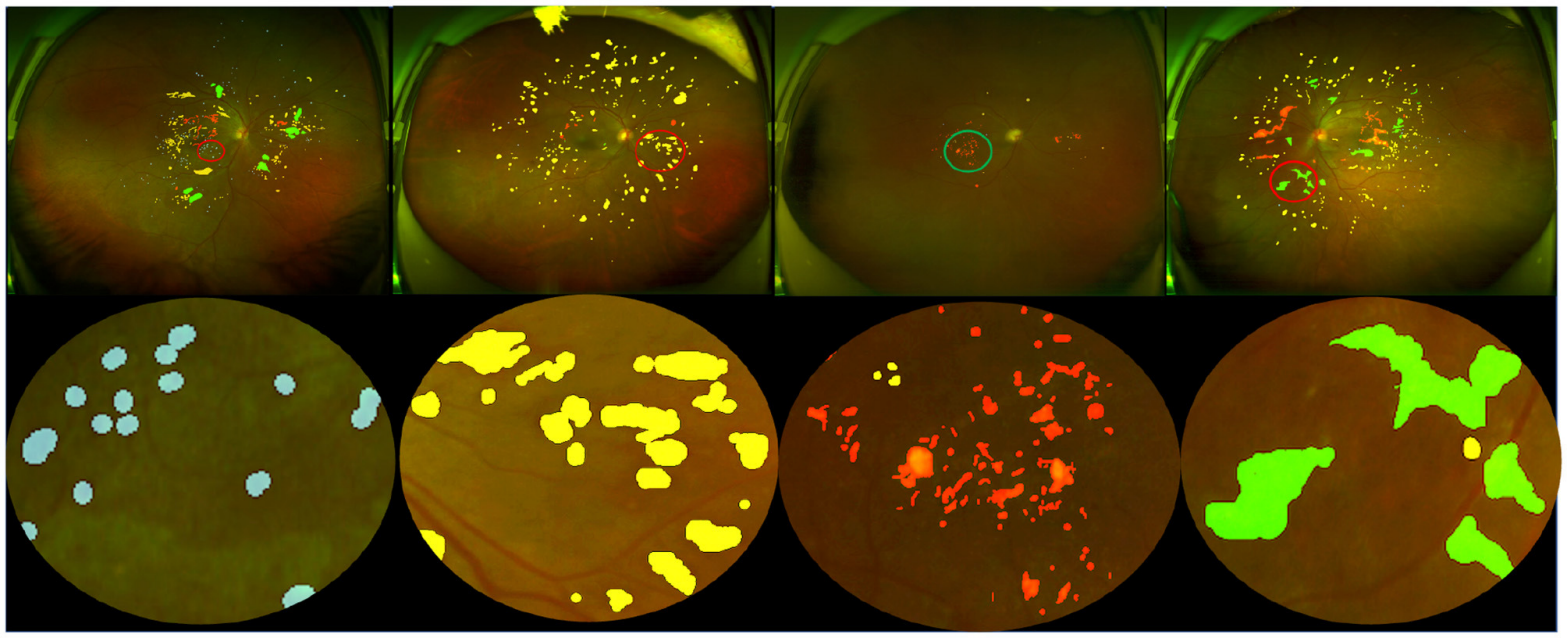
Pixel-level annotation examples from UWF-seg, including four different lesions. The blue, yellow, red, and green denote microaneurysm, hemorrhage, hard exudate, and soft exudate, respectively.

**Dataset annotation:** Lesion annotation in the UWF-seg dataset was conducted using the ITK-SNAP (67) annotation software. The annotations were based on detailed clinical features. Specifically: Microaneurysms (MA) were annotated based on obvious borders and red spots of various sizes distributed at the ends of blood vessels; Hemorrhages (HE) typically manifested as circular or patchy red spots distributed throughout the entire fundus image, often with a relatively large volume; Exudates (EX) were annotated based on their obvious borders and sediment-like appearance, which was relatively small and bright white or yellow-white in color; Soft exudates (SE) usually presented as areas with unclear borders and a fluffy texture, exhibiting a pale white or pale yellow-white color, often growing along the direction of the nerve fiber layer. Partial annotation examples and their corresponding lesion annotations are illustrated in Figure 3. Additionally, DR grading annotations strictly adhered to international DR severity scales.

**Data pre-processing:** The IDRID, EyePACS, DeepDRiD, and Local-UWF datasets exhibit variations in lighting conditions and resolutions. Consequently, a preprocessing method based on Van Grinsven et al. (68) was employed to standardize image quality and enhance texture details. Moreover, to address class imbalance and improve model robustness, horizontal and vertical flipping, along with rotation at consistent angles, were applied to both images and labels. Notably, UWF images often contain structural artifacts like eyelids and eyelashes, which can negatively impact tasks such as lesion segmentation by causing model overfitting. To mitigate this issue, a preprocessing approach similar to that of Ju et al. (7) was adopted. Specifically, U-Net segmentation networks were trained to remove artifacts while preserving essential semantic information. Subsequently, all images underwent the center-cut method to trim the edges of the UWF fundus images.

### 4.2 Evaluation metrics

To quantitatively evaluate the performance of the lesion segmentation task, we compute several metrics including the Dice Similarity Coefficient (Dice), Area Under the Curve of the Receiver Operating Characteristic (AUC-ROC), Area Under the Curve of the Precision-Recall (AUC-PR), and Mean Absolute Error (MAE). The MAE is defined as:


$$\text{MAE}=\frac{\text{1}}{w\times h}\overset{w}{\underset{x}{\sum }}\overset{h}{\underset{y}{\sum }}|M_{i}\left(x,y\right)-Y\left(x,y\right)|$$

where M_*i*_ indicates the final prediction of the DR lesion. To evaluate the performance of DR grading, we utilize several widely-used metrics for multi-class classification, including Accuracy (ACC), Weighted Sensitivity (Sen), Specificity (Spe), and the quadratic weighted kappa metric. The kappa metric is defined as follows:


$$\text{kappa}=\frac{{\text{p}}_{o}-{\text{p}}_{e}}{1-{\text{p}}_{e}}$$

where p_*o*_ and p_*e*_ represent the extent to which raters agree and the expected probability of chance agreement, respectively.

### 4.3 Implementation details

The training methodology for the DeepMT-DR model comprises three stages. In the first stage, we train the auxiliary task subnet, which focuses on UWF lesion segmentation. The primary objective of this stage is to extract adequate pathological features to support the main DR grading task. In the second stage, we pre-train the DR grading subnet using the CFP DR severity classification task to enhance UWF performance. In the third stage, we utilize prior knowledge and the proposed LEAM to fine-tune the DR grading module, leveraging the models pretrained in the first two stages. Furthermore, in all training stages, we optimize the model parameters using the Adam optimizer, augmented with weight decay. Table 2 presents the values assigned to the critical hyper-parameters during the training stages. In our implementation, all images were resized to 512 × 512 pixels. We implemented the proposed networks using Python based on the PyTorch package, and the PC we used contained two GPUs (NVIDIA GeForce GTX 3090 Ti 24GB each).

**Table 2:** Values of some key hyper-parameters in the three training stages.

	**Initial learning rate**	**Weight**	**Batch**
StageI	0.0001	0.0005	8
StageII	0.0005	0.0005	32
StageIII	Same as Stage II		

### 4.4 Lesion segmentation performances

Before quantifying the impact of lesion information on grading performance, we first demonstrate the effectiveness of the adversarial lesion generator based on convolutional modulation for unsupervised segmentation on the UWF-seg dataset. We evaluate two different types of lesions: dark lesions and bright lesions, which are key indicators of diabetic retinopathy (DR), using metrics including Dice similarity coefficient, AUC-ROC, AUC-PR, and mean absolute error (MAE). Dark lesions such as microaneurysms (MA), blot hemorrhages, dot hemorrhages, and flame hemorrhages are clinical signs observed in the early stages of DR. On the other hand, bright lesions such as hard exudates (EX) and soft exudates (SE) are characteristic of more severe stages of the disease. Therefore, detecting both bright and dark lesions without further subdividing them into specific types is sufficient for initial DR grading. We investigate each proposed component of the final model alongside two baselines. **Base1:** The pre-trained base segmentation model is trained in a fully supervised manner using 54 CFP images from IDRID and evaluated using the 27 IDRID test images, aiming to enhance the quality of knowledge learned from the source domain. **Base2:** The pre-trained base segmentation model uses 81 CFP images without an adversarial transfer strategy, and is directly tested on the UWF-seg dataset.

The detailed segmentation performances of these methods are reported in Tables 3, 4. For **Base1**, several metrics such as Dice and AUC-ROC are already comparable to most segmentation models trained on the same data, fully demonstrating that the improved Base possesses good lesion extraction capabilities. For **Base2**, applying the model trained on the source domain directly to the target domain, the Dice value for bright and dark lesions were only 31.8%, 29.5%, respectively, demonstrating a significant domain bias problem between the source and target domain data. On the UWF-seg dataset, a adversarial domain adaptation based UWF lesion segmentation model consistently outperforms Base2. the Dice value for bright and dark lesions increases by 9.8%, 13.4%, respectively, proving that adversarial domain adaptation can indeed benefit the UWF segmentation results. It is worth noting that, for bright lesions, the value of AUC-ROC actually decreased. This may be because AUC-ROC is more sensitive to the classification boundary between positive and negative classes, leading to more mis-classifications on the decision boundary of the classifier. Furthermore, after improving AUC-ROC, the AUC-PR values tend to be generally lower. This is because pathological regions related to DR typically represent only a small portion of the image, while normal regions constitute the vast majority. Consequently, models often predict normal regions more easily while neglecting pathological ones. To address this issue, we can adjust the threshold to strike a balance between the two. With the CMFF design, which exploits more contextual information to improve the identification of complex lesions, a clear improvement is observed. Specifically, significant improvements were observed for dark lesions, with an average gain of 2.2% for the Dice value.

**Table 3:** Comparison of unsupervised segmentation of bright lesion based on convolutional modulation adversarial lesion generators.

**Lesion**	**Bright lesion (EX+SE)**			
**Methods**	**Dice**	**AUC-ROC**	**AUC-PR**	**MAE**
Base1	0.647 + 0.121	0.989 + 0.008	0.712 + 0.143	0.011 + 0.010
Base2	0.318 + 0.165	0.976 + 0.024	0.289 + 0.214	0.010 + 0.003
Base2+Adv	0.416 + 0.166	0.950 + 0.056	0.381 + 0.209	0.004 + 0.002
Base2+Adv+CMFF	0.417 + 0.161	0.970 + 0.032	0.443 + 0.203	0.006 + 0.003

**Table 4:** Comparison of unsupervised segmentation of dark lesion based on convolutional modulation adversarial lesion generators.

**Lesion**	**Dark lesion (MA+HE)**			
**Methods**	**Dice**	**AUC-ROC**	**AUC-PR**	**MAE**
Base1	0.522 + 0.127	0.963 + 0.037	0.544 + 0.175	0.013 + 0.018
Base2	0.295 + 0.181	0.890 + 0.060	0.289 + 0.203	0.029 + 0.021
Base2+Adv	0.429 + 0.150	0.906 + 0.055	0.435 + 0.199	0.015 + 0.014
Base2+Adv+CMFF	0.451 + 0.154	0.903 + 0.053	0.446 + 0.192	0.017 + 0.017

**Figure 4:**
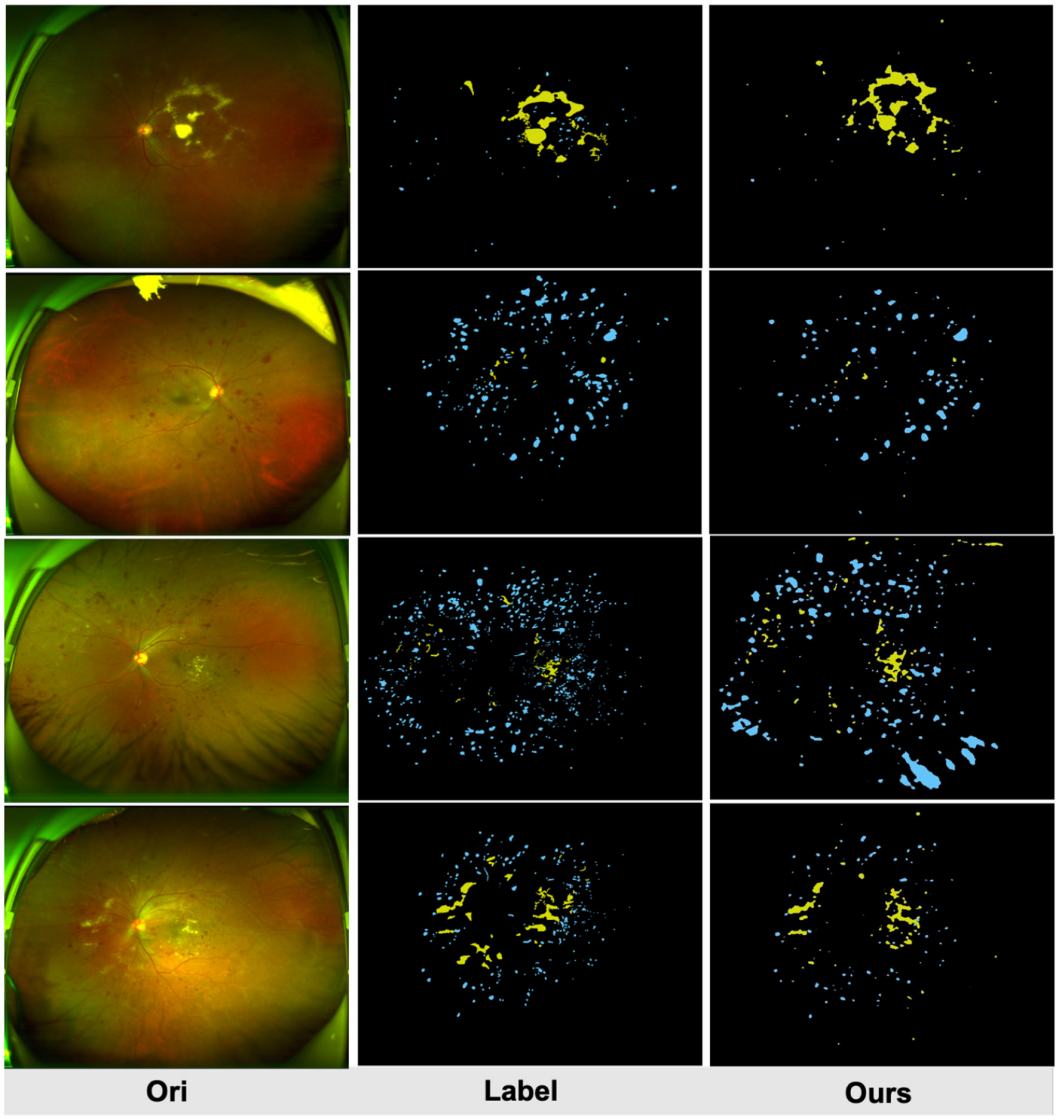
Qualitative multi-lesion segmentation results. Yellow and blue represent light and dark lesions, respectively.

Figure 4 compares the subjective segmentation results of two different lesions for the pre-trained lesion segmentation model adopting the limited UWF data. As seen, the lesion segmented masks by our method are more close to the ground-truth.

### 4.5 DR grading performances

The proposed method was first compared the following four representative types of UDA methods which were designed for classification. These methods include: Domain Separation Networks (**DSN**) (69), Adversarial Discriminative Domain Adaptation (**ADDA**) (70), Maximum Classifier Discrepancy (**MCD**) (71), Dynamic Weighted Learning (**DWL**) (72), and the (**ULTRA**) (26), As in the top half of Table 5. Note, ULTRA is a model specifically proposed for DR grading in UWF image. Furthermore, although our approach is unsupervised, fully supervised training can also be performed when the labels of the UWF images are available, which we define as Ours^⋆^. So, we also compared the proposed method to the state-of-the-art deep-learning-based methods for UWF image DR classification, for example, **VGG-16** (73), **ResNet50** (62), and **CycleGAN** (7). Notably, **CycleGAN** method is the only method that uses CFP images to aid the training of UWF images. As in the lower part of Table 5.

**Table 5:** The DR grading results over the Local-UWF dataset.

**Methods**	**Acc**	**PRE**	**F1**	**Kappa**
DSN (69)	0.5027	0.3582	0.6097	0.3287
ADDA (70)	0.5396	0.5513	0.5447	0.4142
MCD (71)	0.5523	0.4816	0.5377	0.4874
DWL (72)	0.5646	0.5160	0.6049	0.4282
ULTRA (26)	0.5832	0.5210	0.5518	0.4903
VGG-16 (73)	0.6417	0.6496	0.6411	0.5370
Resnet50 (62)	0.6563	0.6423	0.6734	0.5478
CycleGAN^*^ (7)	0.6292	0.6278	0.6389	0.5159
Ours	0.5912	0.5240	0.6423	0.4648
Ours^*^	0.6813	0.6743	0.6889	0.5861

^*^Indicates the method is fully supervised, i.e., the grading labels of UWF images are used in the training phase.

#### 4.5.1 Classification performance of local-UWF

In general, deep learning methods trained in a fully supervised manner tend to yield superior classification results compared to unsupervised DA methods, and the difference in performance is relatively significant. This fact further underscores the significant challenges associated with leveraging CFP images to aid in the diagnosis of UWF images. However, the proposed method outperforms these UDA methods in most metrics.

For example, our method demonstrates a significant advantage over the **DWL** method, with an increase in accuracy and *Kappa* of approximately 2.66% and 3.66%, respectively. Furthermore, despite incorporating a reconstruction loss in the DSN method to capture more generalized features, this also introduces a tendency for the model to disregard image-specific details, such as lesions present in CFP and UWF images, resulting in suboptimal performance of the DSN approach for this particular task. When trained in a supervised manner, most of the models perform well, demonstrating the feasibility of grading UWF images with DL methods. Compared with the state-of-the-art deep learning method, Ours^⋆^ demonstrated competitive performance across all metrics. For example, our method exhibits a significant advantage over the **CycleGAN** method, with increases in accuracy, precision, F1 score, and Kappa of approximately 5.21%, 4.65%, 4.99%, and 7.02%, respectively. The main reason for this is that the **CycleGAN** method generates UWF images from CFP images by style transfer, and the performance of the grading model depends on the quality of the synthesized images.

**Figure 5:**
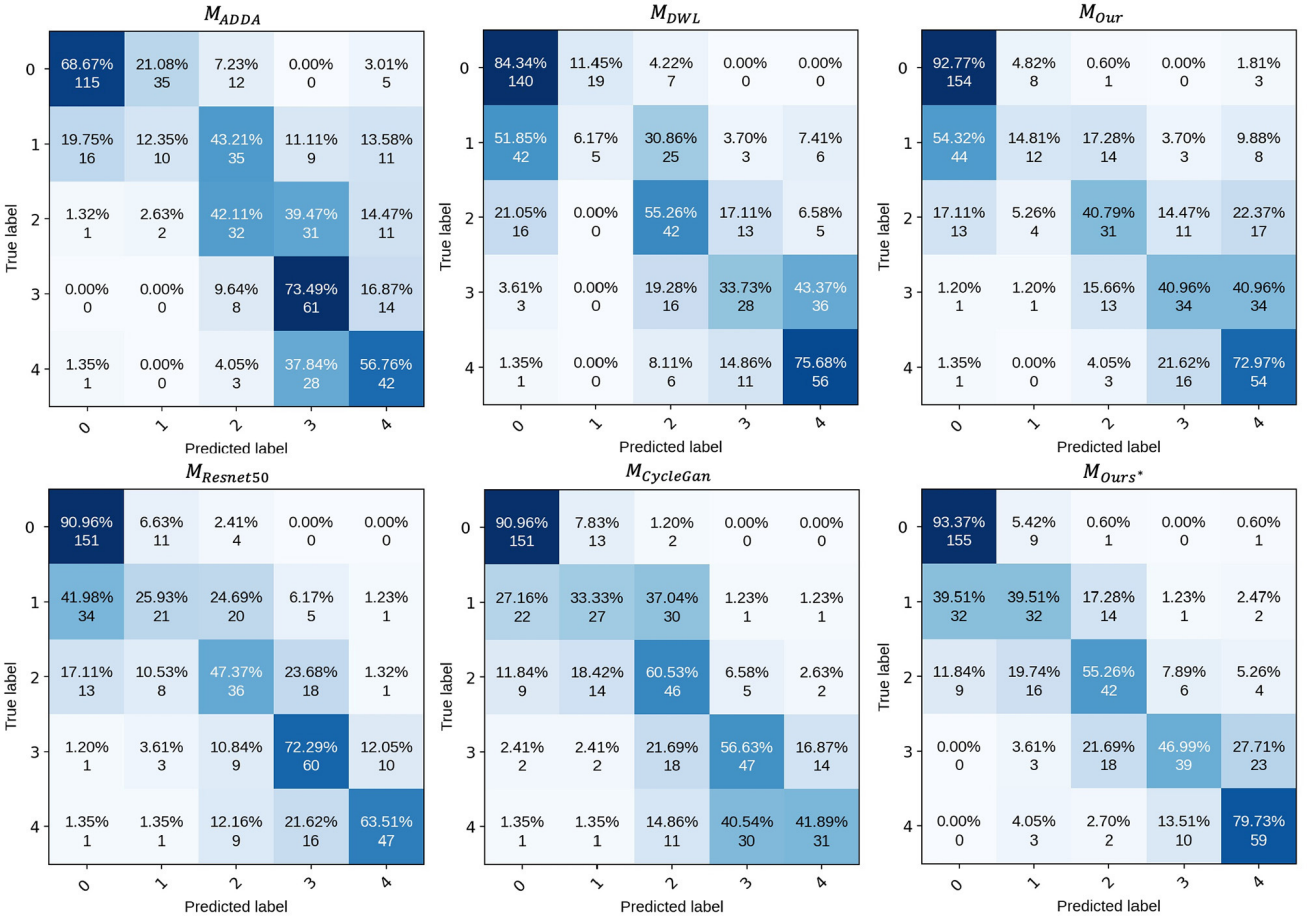
Confusion matrix of proposed model.

To analyze the performance of the proposed model for UWF DR grading, we have provided the confusion matrix in Figure 5. This matrix displays the recognition results of the model across different categories. Overall, the proposed model performs well in all classes except for class 1.

### 4.6 Ablation study

In this section, we perform an ablation study to analyze the effectiveness of each key component. Our Net employs three main components to form its classification framework: unsupervised DR grading module, adversarial lesion segmentation module and Lesion external attention module, so we analyze and discuss the network under different scenarios to validate the performance of each key component of our model. The results of different combinations of these modules are reported in Table 6.

**Table 6:** Performance comparisons of ablation studies.

**Method**	**Training**	**Resnet-50**	**C1+C2+D**	**Lesion**	**LEAM**	**ACC**	**PRE**	**F1**	**Kappa**
*M*_CFP_	*C*_*label*_	✓				0.2647	0.4397	0.2786	0.1057
*M*_Transfer_	*C*_*label*_/*U*_*unlabel*_	✓	✓			0.5458	0.5340	0.5955	0.4069
*M*_Lesion_	*C*_*label*_/*U*_*unlabel*_	✓	✓	✓		0.5563	0.4493	0.6262	0.4001
*M*_Ours_	*C*_*label*_/*U*_*unlabel*_	✓	✓	✓	✓	0.5912	0.5240	0.6423	0.4648

*C* and *U* denotes the CFP and UWF datasets, respectively.

#### 4.6.1 The effectiveness of unsupervised DR grading module

To explore the impact of the UDA DR grading sub-network, we employed a ResNet-50 grading model as the backbone, denoted as *M*_*CFP*_, which was trained solely on the EyePACS subset and tested on the UWF dataset. It's important to note that the backbone model achieves an accuracy of 26.47% (as shown in Table 6), indicating the significant domain gap between CFP images and UWF images.

Furthermore, we explored the C1+C2+D method, which involves joint training using both CFP and UWF images with UDA techniques. Encouragingly, this method outperformed the *M*_CFP_ backbone model, demonstrating significant improvements across several indicators. This result underscores the effectiveness of leveraging UDA to jointly train CFP and UWF images, thereby reducing domain discrepancies and enhancing the accuracy of DR grading. By leveraging the complementary information from both CFP and UWF domains, our approach showcases its efficacy in achieving superior performance in DR grading tasks. These findings underscore the potential of UDA techniques and the integration of diverse image sources for enhancing the accuracy and reliability of DR grading models.

#### 4.6.2 The effectiveness of adversarial lesion segmentation module

As described in Section 3.3, a pivotal component of our proposed method is the adversarial lesion segmentation module, aimed at capturing multi-lesion features from annotated UWF images. This addresses the challenge of lacking prior guidance during the decision-making stage of DR. Detailed ablation results for the adversarial lesion segmentation module are presented in Section 4.4. Specifically, we observe an increase of approximately 1.05% in accuracy (ACC) for *M*_Lesion_ compared to *M*_Transfer_. This suggests that the lesion generation module provides additional lesion information, and the specific lesion features are beneficial for distinguishing DR subtypes, aligning with the findings of epidemiological studies.

#### 4.6.3 The effectiveness of the LEAM

In Section 3.3.3, we introduced the incorporation of fully integrated lesion features into our approach. To ascertain the effectiveness of the Lesion External Attention Module (LEAM), we compared the performance of the model with and without LEAM, denoted as *M*_Lesion_ and *M*_Ours_ respectively. The results demonstrated that the feature fusion strategy facilitated by LEAM significantly enhances the classification performance, with a 3.49% increase in accuracy (ACC) and a 6.48% increase in kappa. This observation suggests that the proposed LEAM effectively embeds lesion-specific knowledge into the grading module. By focusing attention on salient lesion features, LEAM facilitates the extraction and integration of crucial information, thereby improving the overall capability of the grading model to accurately classify retinal images.

## 5 Discussions and conclusion

Several existing studies have highlighted the significant advantages of ultra-widefield (UWF) imaging over color fundus photography (CFP) in monitoring diabetic retinopathy (DR) progression. However, due to limited datasets and annotations, the field of UWF-based DR-assisted diagnosis remains relatively unexplored. Moreover, most existing studies utilizing UWF images and deep learning methods for DR diagnosis employ end-to-end models lacking guidance from prior knowledge and interpretability in decision-making.

In this study, we introduce a deep learning-based method aimed at robust predictions for DR in UWF photography, focusing on unsupervised lesion-aware domain adaptation. However, achieving robust predictions for DR in an unsupervised manner presents two significant challenges: Firstly, overall metrics for segmenting UWF lesions need improvement, and there is a lack of detailed class information; secondly, lesion segmentation and disease grading are separate tasks requiring individual attention and improvement.

The main contribution of our work lies in accomplishing the tasks of lesion segmentation and automatic grading of DR using CFP images to assist UWF image analysis through the innovative application of unsupervised domain adaptation (UDA) methods. We aim to incorporate clinical priors into the deep learning algorithm through lesion segmentation of UWF images and the explicit utilization of light-dark lesion data to enhance DR classification accuracy. Our ablation study demonstrates the effectiveness of our specifically designed components.

### 5.1 Limitations

#### 5.1.1 The performance of UWF segmentation network needs to be improved

**Figure 6:**
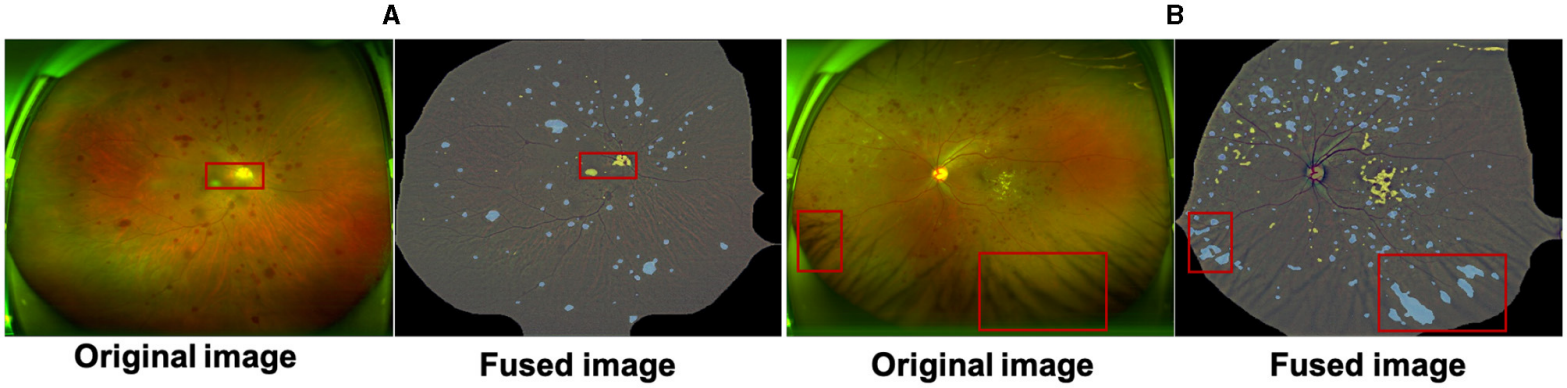
The macula and optic cup in **(A)** are incorrectly identified as bright lesions; the intersection of the eyelashes and fundus in **(B)** is also incorrectly detected as a dark lesion. All samples are a fusion of the pre-processed maps with the results of the lesion features.

In this work we proposes a UWF lesion segmentation network based on adversarial domain transfer, which simulates the process of clinical doctors diagnosing DR based on detailed lesion features. Although this method achieved certain segmentation results on the UWF-seg dataset, the overall performance still needs to be improved. UWF images are often obstructed by eyelids and eyelashes, and these artifacts may affect the screening performance of models trained on clean images. Although pre-processing can remove some artifacts, it also masks useful information in the surrounding area and there are still some false positives cases. As Figure 6A shows, the macula and the optic disc will be wrongly detected as bright lesions, where Figure 6B shows that the intersection of eyelashes and fundus will also be wrongly segmented as a lesion area. Therefore, an effective method for removing UWF image artifacts while preserving key structures is urgently needed. In addition, the irregular shape of lesions, their similarity to surrounding normal tissues, and mutual occlusion make them difficult to segment correctly using unsupervised methods. To overcome these challenges, future research can adopt deep reinforcement learning or semi-supervised training to improve the model's segmentation ability for complex lesions.

#### 5.1.2 Collaborative training framework needs to be developed

In this work, we propose an ULTRA (Unsupervised Lesion Transfer Learning for Disease Recognition and Assessment) network based on UWF images for automatic grading of diabetic retinopathy (DR), and its effectiveness has been demonstrated through extensive experiments. However, our approach treats lesion segmentation and disease diagnosis as separate tasks and combines their features using a specific fusion strategy. This requires manual selection of fusion strategies and hyperparameter tuning, potentially resulting in information loss in the fusion process.

To address this limitation, future research could explore the development of a collaborative training framework and optimize joint training strategies to ensure the accuracy of both lesion segmentation and disease diagnosis. By enhancing the effectiveness of joint learning, such efforts can lead to improved performance and reliability in automated DR grading systems based on UWF images.

### 5.2 Analysis on failure cases

**Figure 7:**
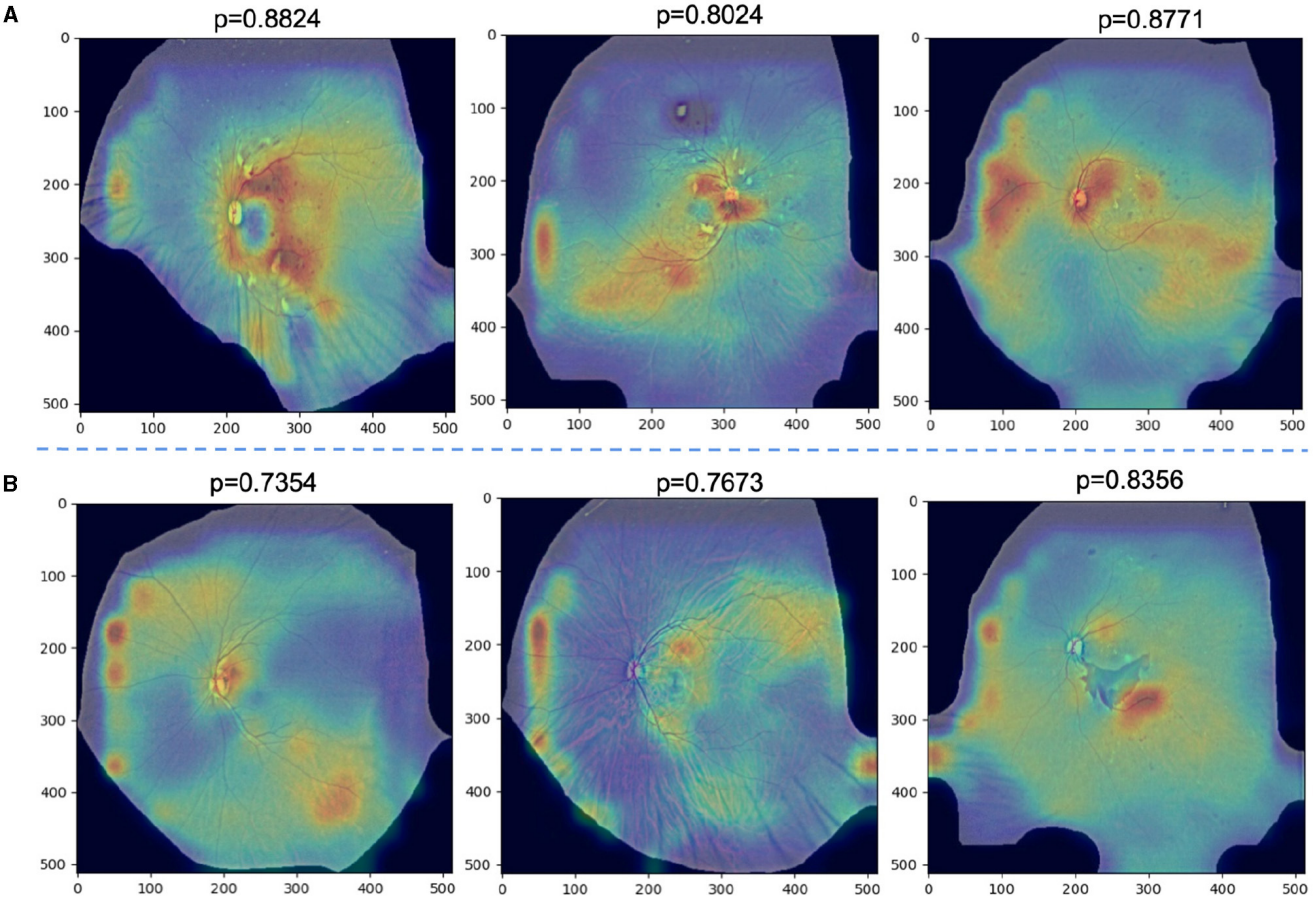
**(A)** Examples of successfully ignored the artifact model and instead focused on specific lesions and correctly identified the degree of DR severity with a high confidence rate. **(B)** Examples of misclassifications.

We further analyze the failed classification cases by GradCAM. Specifically, Figure 7A demonstrates successful predictions of DR severity grading by the model, while Figure 7B displays examples of misclassifications. All images are preprocessed and overlaid with heatmaps. it is observed that in Figures 4–6A, despite the presence of interfering factors such as eyelash artifacts, ULTRA consistently disregards these artifacts and focuses primarily on lesion information, resulting in accurate predictions of DR severity with high confidence. Based on our observation on cases shown in Figure 7A, we found that proposed model pays more attention to lesion information, despite the presence of interfering factors such as eyelash artifacts, resulting in accurate predictions of DR severity with high confidence. However, at times, these interfering factors can cause confusion, as evident in Figure 7B. These misclassifications typically occur in the No DR or NPDRI stages, where the model lacks sufficient reliable attention and tends to prioritize peripheral artifacts, mistakenly identifying them as lesions, particularly in the vicinity of eyelashes. Notably, in the first example, the optic disc may be misinterpreted as exudates or a large hemorrhage, and the intersection between the eyelashes and eyelid is incorrectly identified, leading to the erroneous classification of the case as NPDRIII instead of No DR. In the third example, a PDR image is incorrectly diagnosed as NPDRIII primarily due to the failure in accurately identifying the patchy hemorrhage in the image. It also shows that our proposed use of a lesion prior as one of the classification features is feasible, and there is reason to believe that as lesion performance improves in future work, our model will be able to more accurately identify the degree of DR severity.

### 5.3 Conclusion

In this work, we designed a specific approach and strategies to solve the above mentioned issues. Specifically, we proposed a novel DR grading network for unsupervised lesion-aware domain adaptation in UWF images. Our approach tackles the task of grading DR by leveraging unsupervised domain adaptation techniques while explicitly considering the presence of lesions. By incorporating lesion-specific knowledge into the model, we aimed to improve its ability to generalize across different domains and accurately grade UWF images. To achieve this, we developed a comprehensive framework that combines DA strategies with lesion-aware mechanisms. By leveraging unsupervised learning techniques, our approach can effectively adapt the grading model from a source domain (e.g., CFP images) to a target domain (e.g., UWF images) without the need for labeled data in the target domain. Moreover, our framework incorporates lesion-aware mechanisms, such as the Lesion Embedding Attention Module (LEAM), to ensure that the model can effectively capture and exploit the discriminative information present in lesion regions. By integrating these novel components and adopting a holistic approach, our proposed method aims to address the challenges associated with domain shift and the unique characteristics of UWF images in DR grading. Through experimental evaluations and comparisons, we demonstrate the effectiveness and superiority of our approach in accurately grading UWF images, thus contributing to improved diagnosis and management of diabetic retinopathy.

## Data availability statement

Publicly available datasets were analyzed in this study. This data can be found here: https://www.kaggle.com/c/diabetic-retinopathy-detection/overview.

## Ethics statement

The studies involving humans were approved by Eye Pricture Archive Communication System. The studies were conducted in accordance with the local legislation and institutional requirements. The participants provided their written informed consent to participate in this study.

## Author contributions

TC: Writing – original draft, Writing – review & editing, Supervision, Validation. YB: Writing – original draft, Writing – review & editing, Data curation, Software. HM: Writing – original draft, Writing – review & editing, Project administration. SL: Writing – original draft, Writing – review & editing, Formal analysis. KX: Writing – original draft, Writing – review & editing, Visualization. ZX: Writing – original draft, Writing – review & editing, Funding acquisition. SM: Writing – original draft, Writing – review & editing, Formal analysis. FY: Writing – original draft, Writing – review & editing. YZ: Writing – original draft, Writing – review & editing.

## Footnotes

^1^ https://www.kaggle.com/c/diabetic-retinopathy-detection/
